# Comparative genomic analysis suggests that the sperm-specific sodium/proton exchanger and soluble adenylyl cyclase are key regulators of CatSper among the Metazoa

**DOI:** 10.1186/s40851-019-0141-3

**Published:** 2019-07-26

**Authors:** Francisco Romero, Takuya Nishigaki

**Affiliations:** Departamento de Genética del Desarrollo y Fisiología Molecular, Instituto de Biotecnología. Universidad Nacional Autónoma de México (IBT-UNAM). Av. Universidad 2001, Col. Chamilpa, 62210 Cuernavaca, Morelos Mexico

**Keywords:** CatSper, Sperm-specific Na^+^/H^+^ exchanger (sNHE), *SLC9C*, Soluble adenylyl cyclase (sAC), *ADCY10*, Testis-specific Na^+^/H^+^ exchanger (NHA), *SLC9B*, Coevolution, Flagellar beat regulation

## Abstract

**Background:**

CatSper is a sperm-specific calcium ion (Ca^2+^) channel, which regulates sperm flagellar beating by tuning cytoplasmic Ca^2+^ concentrations. Although this Ca^2+^ channel is essential for mammalian fertilization, recent bioinformatics analyses have revealed that genes encoding CatSper are heterogeneously distributed throughout the eukaryotes, including vertebrates. As this channel is activated by cytoplasmic alkalization in mammals and sea urchins, it has been proposed that the sperm-specific Na^+^/H^+^ exchanger (sNHE, a product of the *SLC9C* gene family) positively regulates its activity. In mouse, sNHE is functionally coupled to soluble adenylyl cyclase (sAC). CatSper, sNHE, and sAC have thus been considered functionally interconnected in the control of sperm motility, at least in mouse and sea urchin.

**Results:**

We carried out a comparative genomic analysis to explore phylogenetic relationships among CatSper, sNHE and sAC in eukaryotes. We found that sNHE occurs only in Metazoa, although sAC occurs widely across eukaryotes. In animals, we found correlated and restricted distribution patterns of the three proteins, suggesting coevolution among them in the Metazoa. Namely, nearly all species in which CatSper is conserved also preserve sNHE and sAC. In contrast, in species without sAC, neither CatSper nor sNHE is conserved. On the other hand, the distribution of another testis-specific NHE (NHA, a product of the *SLC9B* gene family) does not show any apparent association with that of CatSper.

**Conclusions:**

Our results suggest that CatSper, sNHE and sAC form prototypical machinery that functions in regulating sperm flagellar beating in Metazoa. In non-metazoan species, CatSper may be regulated by other H^+^ transporters, or its activity might be independent of cytoplasmic pH.

**Electronic supplementary material:**

The online version of this article (10.1186/s40851-019-0141-3) contains supplementary material, which is available to authorized users.

## Background

The calcium ion (Ca^2+^) is the fundamental factor that regulates flagellar beating patterns [1]. In mammals, the sperm-specific Ca^2+^ channel CatSper [2] is the main Ca^2+^ regulator of cytosolic Ca^2+^ concentration ([Ca^2+^]_c_) [3]. CatSper is localized on the plasma membrane of the principal piece of the flagellum, and is essential for hyperactivated flagellar motility, an especially vigorous movement of the flagellum required to fertilize the egg [4, 5]. This Ca^2+^ channel is a moderately voltage-dependent channel, and its activity is highly up-regulated by cytoplasmic alkalization [3, 6, 7]. Unlike other voltage-gated Ca^2+^ channels (Ca_v_s), this sperm channel is constituted by four pore-forming α subunits [8] and five additional auxiliary subunits β, γ, δ, ε, and ζ [9–12]. All α subunits and the δ subunit are indispensable for the structure and function of the CatSper channel, because the male gene knock-out mice for each of these subunits are infertile and lack CatSper activity [11, 13].

Despite the essential function of CatSper in mammals, genes encoding CatSper are widely but heterogeneously distributed in all lineages of eukaryotes [14–17]. For example, CatSper is highly conserved in mammals, but the genes encoding CatSper have degenerated to pseudogenes in birds (chicken and zebra finch) [16].

Besides Ca^2+^, cytoplasmic pH (pH_c_) and cyclic adenosine monophosphate (cAMP) are also important factors for regulating flagellar motility [18]. These factors modulate CatSper activity, at least in mouse and sea urchin spermatozoa [3, 19, 20]. Mammalian spermatozoa possess a sperm-specific Na^+^/H^+^ exchanger (sNHE, the product of *SLC9C*) [21] and a bicarbonate-sensitive soluble adenylyl cyclase (sAC, the product of *ADCY10*) [22, 23]. Both proteins are essential for normal sperm function because knock-out mouse models for each gene exhibit male infertility [21, 24, 25]. In contrast to the other NHEs found in somatic cells, sNHE has been proposed to be regulated by membrane potential (Vm) and cyclic nucleotides through a voltage-sensor domain (VSD) and a cyclic nucleotide-binding domain (CNBD), respectively. This proposal was recently proved with sea urchin sNHE expressed in Chinese hamster ovary CHO cells [26]. Cai and Clapham (2008) mentioned (without presenting any evidence) that sNHE shows a similar lineage-specific gene loss as that found for CatSper [16]. Orthologues of the gene encoding human sAC are present in mammals, but are absent from the genomes of *Drosophila melanogaster*, *Caenorhabditis elegans* and plants [27], which corresponds to the distribution of CatSper [16]. In addition, it has been reported that sNHE interacts with the full-length isoform of sAC (sAC_f_), stabilizing both proteins [28]. In fact, in spermatozoa from the sNHE-null mouse, the sAC_f_ isoform was not detected in mature cells and the activity of sAC was significantly diminished [28]. Moreover, the suppressed sperm motility in sNHE-null spermatozoa can be rescued by membrane-permeable cAMP analogues [21] or by the production of cAMP through a photo-activated AC (bPAC) [29] rather than alkalization of pH_c_ by NH_4_Cl [21]. Together, these previous findings suggest that there is a functional coupling among CatSper, sNHE, and sAC. In the present study, we explored the distribution of genes encoding CatSper, sNHE and sAC across eukaryotes to find coevolutionary linkages among these sperm components.

## Methods

### Database mining and species selection

To determine the presence of CatSper, sNHE and sAC along the entire phylogeny of eukaryotes, we followed the methodology previously described by Cai and Clapham [16]. In brief, we performed TBlastN and BlastP searches [30, 31] with the protein sequences of *Homo sapiens* (Human), *Strongylocentrotus purpuratus* (purple sea urchin) and *Exaiptasia pallida* (brown glass anemone) as queries against the respective genomic databases (Additional file 1: Table S1).

To define species that conserve CatSper, sNHE or sAC, we applied the following criteria. For CatSper, a positive case should have at least four α pore-forming subunits as in the mouse [13]. In the case of sNHE, we considered species conserving at least one orthologue of the *SLC9C* gene family as being positive. We defined only NHEs containing both VSD and CNBD as orthologues of SLC9C and those preserving only CNBD as homologues of sNHE. For sAC, proteins possessing two catalytic domains without transmembrane segments were selected as candidates of sAC. Furthermore, those conserving a P-loop nucleoside triphosphate hydrolase (NTPase) domain [32] were defined as an sAC, although the exact function of this domain remains unknown.

#### Chromosome synteny and pseudogene identification

To evaluate synteny block conservation corresponding to *SLC9C, ADCY10* and *SLC9B* (a gene encoding testis-specific NHE named NHA), we used the Genomicus browser (http://genomicus.biologie.ens.fr) [33]. We also used the Ensembl genome browser (http://www.ensembl.org/) [34, 35] to identify the flanking genes of *SLC9C, ADCY10* and *SLC9B*.

Furthermore, to determine the presence of a degraded *SLC9C* sequence, we acquired two genomic fragments; one flanked by *GAL* and *PPP6R3* in a chromosome of the white-throated tinamou (*Tinamus guttatus*) and the other (non-coding RNA) localized downstream of *FUS* in FlyBase (DmeI\asRNA:CR45143-RA). Thereafter, both sequences were processed using Small Exon Finder (http://gander.wustl.edu/~wilson/smallexonfinder/index.html) [36] to obtain the deduced amino acid sequence from the predicted exons. Each deduced amino acid sequence was aligned with that of SLC9C of phylogenetically close organisms. Alignments were performed using ClustalW with its default parameters [37], and the visualization was prepared with the R package msa (https://bioconductor.org/packages/release/bioc/html/msa.html) [38]. Abbreviations for flanking genes are listed in (Additional file 2: Table S2).

#### Phylogenetic analysis

To perform molecular phylogenetic analysis for sAC and sNHE, we selected at least one protein sequence from each taxonomic group as a representative, and finally used 33 annotated protein sequences for sAC and 26 protein sequences for sNHE. The GenBank access ID sequences we used for the analyses are summarized in Additional file 3: Table S3. For sAC, we included the *Dictyostelium* homologue of mammalian sAC, which encodes a soluble guanylyl cyclase (sGC-Amoeba) [39]. Phylogenetic reconstructions were conducted in MEGA7 [40] by Maximum Likelihood, using the most suitable substitution model for each alignment: the Whelan And Goldman + Freq. model [41] for sAC and the Le_Gascuel_2008 model [42] for sNHE. Initial trees for the heuristic search were obtained by applying the Neighbour-Joining method to a matrix of pairwise distances estimated using the JTT model. All positions with less than 80% site coverage were trimmed.

#### Motif and domain analysis in sAC sequences

To determine domain variance among sAC homologue sequences, SUPERFAMILY analysis (http://supfam.org/SUPERFAMILY/) was performed using the 33 amino acid sequences detailed in Additional file 3 Table S3 [43, 44]. In the same way, we performed a Multiple EM for Motif Elicitation (MEME) analysis (http://meme-suite.org) to determine metazoan-specific motifs [45]. We delimited the search to a maximum of 20 motifs with a zero or one per sequence occurrence of a length of 60–200 amino acids. Both analyses were depurated manually.

#### Statistical analysis for coexistence relationship among CatSper, sNHE and sAC

**Fig. 1:**
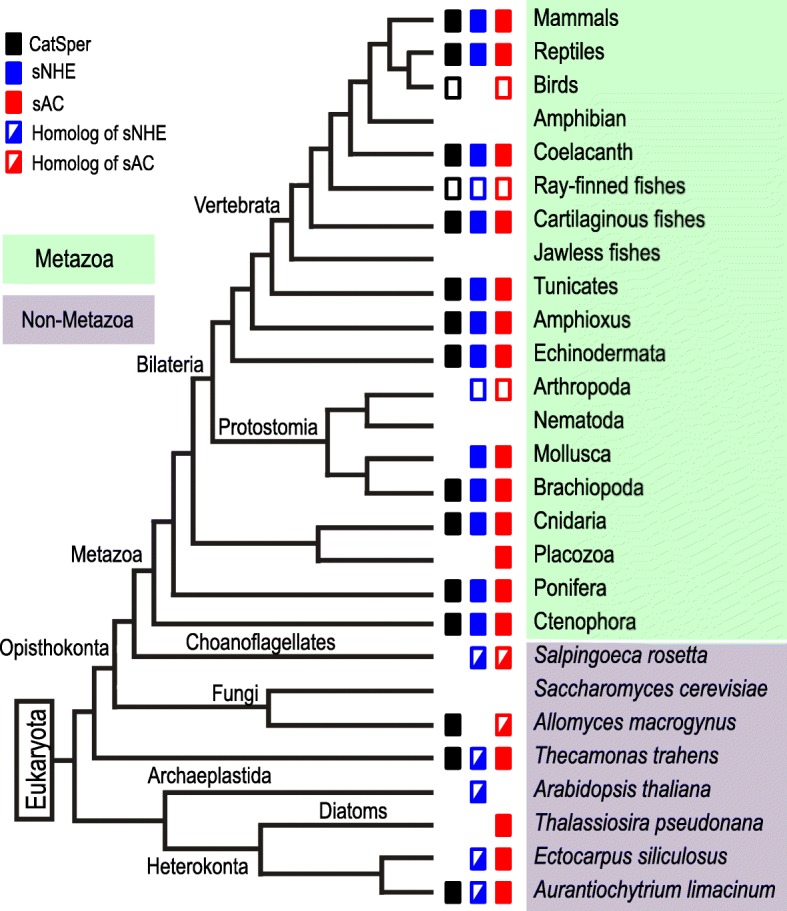
Distribution of genes encoding CatSper, sNHE, and sAC in the entire phylogenetic tree of eukaryotes. The tree indicates the metazoan taxonomic groups (green background) and non-metazoan species (purple background). Closed boxes represent the presence of genes encoding each protein: CatSper (black), sNHE (blue) and sAC (red). Taxonomic groups with open boxes have varied gene distributions within the groups (the detailed distributions can be seen in the following figures). Homologues for sNHE and sAC are represented as half-filled boxes, blue and red, respectively. The phylogenetic tree was prepared based on the Tree of Life Web project (http://www.tolweb.org/tree/) with some modifications based on recent reports [93–96]. The branching patterns do not represent the proportional evolutionary rate

**Fig. 2:**
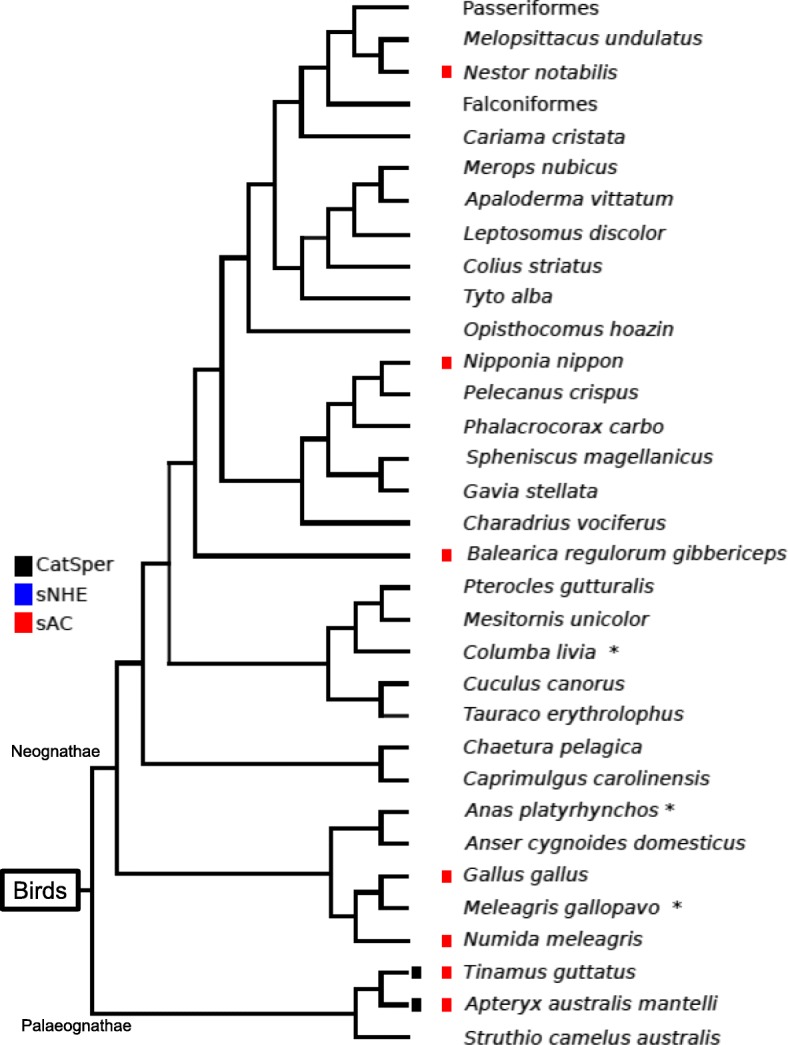
Distribution of genes encoding CatSper and sAC in birds. Only two species belonging to the Palaeognathae show conservation of genes encoding CatSper (black boxes), and no birds show conservation of sNHE. In some species, full or truncated isoforms of sAC (red box) are conserved. Species marked with an asterisk possess a pseudogene for the sAC (Fig. 5C). This phylogenetic tree was prepared based on a recent phylogenetic study of birds [97]. The branching patterns do not represent the proportional evolutionary rate

**Fig. 3:**
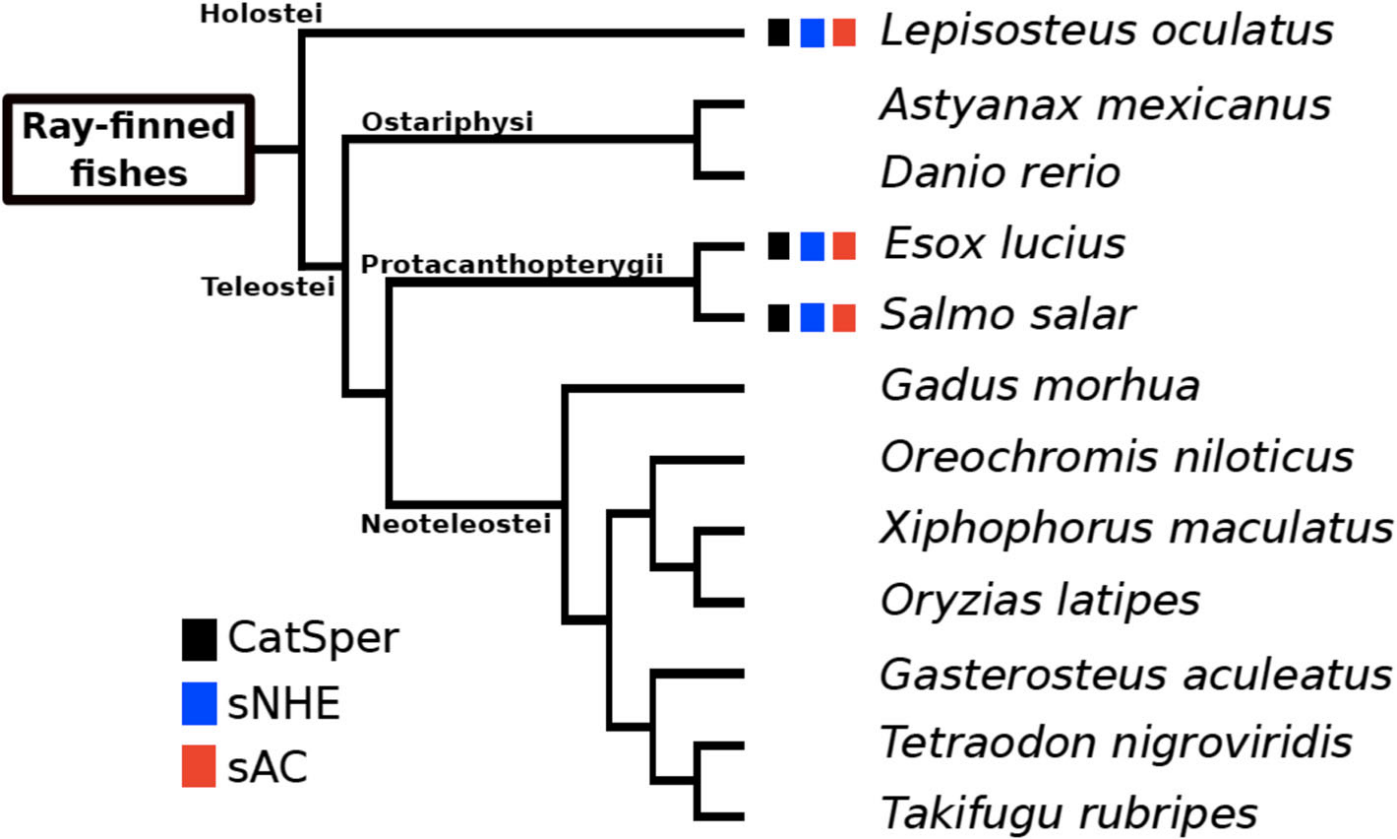
Distribution of genes encoding the three proteins in 12 species of ray-finned fishes. Boxes represent the presence of genes as shown in Fig. 1. The phylogenetic tree was prepared based on a recent phylogenetic study of this taxon [93]. The branching patterns do not represent the proportional evolutionary rate

**Fig. 4:**
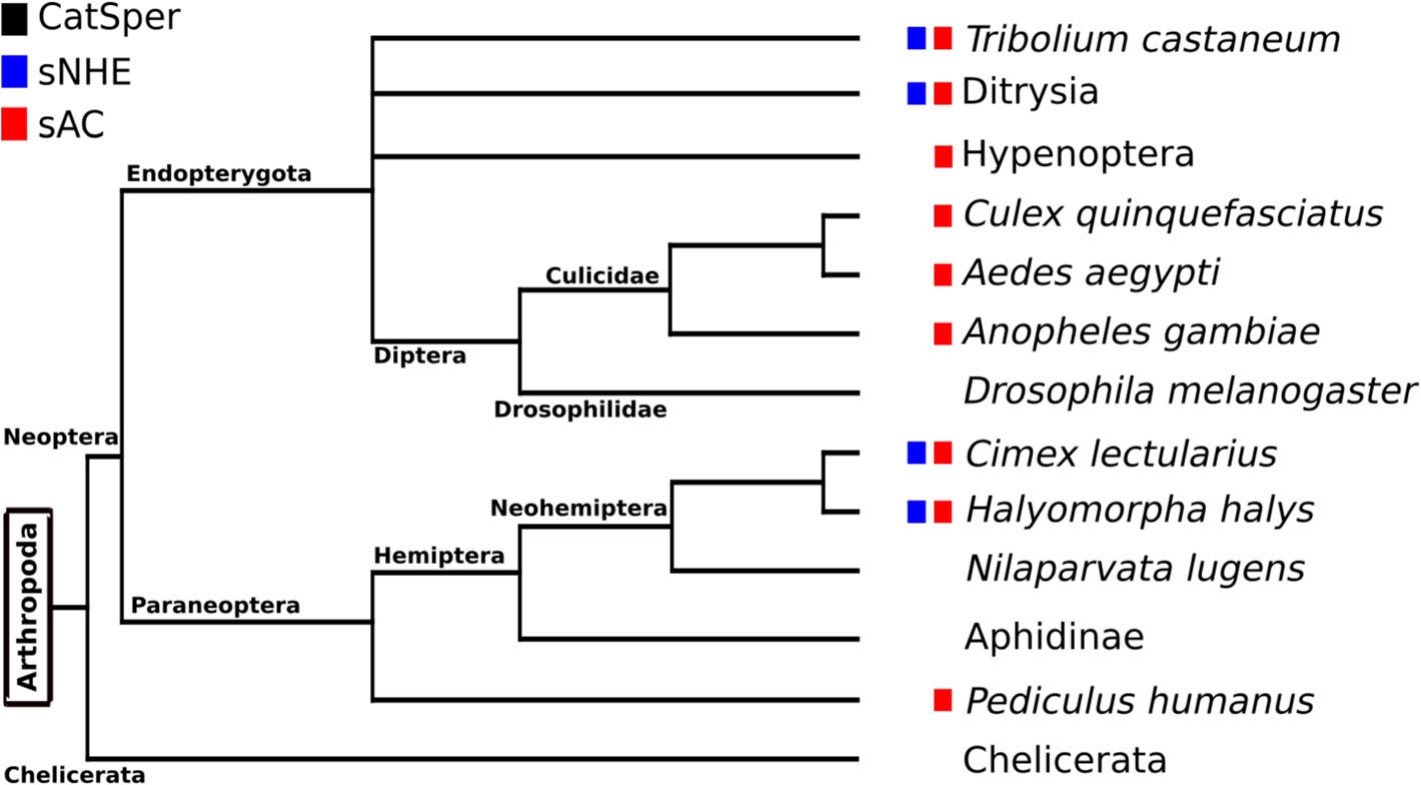
Distribution of genes encoding the three proteins in the Arthropoda. Boxes represent the presence of genes as shown in Fig. 1. The phylogenetic tree was prepared based on the Tree of Life Web project (http://www.tolweb.org/tree/). The branching patterns do not represent the proportional evolutionary rate

To obtain the relationship among the three proteins from the point of view of correlated distribution, we first examined the data illustrated in Figs. 1-4 and counted the fewest events for any loss of gene (or genes) in the Metazoa, as shown in Additional file 7 Figure S4 Based on these gene-loss patterns, we constructed a binary code table (Additional file 8 Figure S5A) and tested for associations between paired events by applying Pearson’s product moment correlation coefficient [46]. We also compared the correlation coefficient for each protein against a randomized variable 500,000 times using R [47]

## Results

### Coevolution of sNHE and sAC together with CatSper in Metazoa

The distribution of genes encoding CatSper, sNHE and sAC across the eukaryotes is summarized in Fig. 1. Because we found heterogeneous distribution patterns for birds, ray-finned fishes, and arthropods, these are indicated as having an ambiguous classification (open boxes; Fig. 1). The detailed gene distribution for each individual taxonomic group (or subtaxon) is shown in separate figures: Figs. 2, 3, and 4 for birds, ray-finned fishes, and arthropods, respectively. It was reported previously that the genes encoding CatSper can be found in all phylogenetic groups of eukaryotes [14]. In contrast, our analysis indicates that sNHE is conserved exclusively in Metazoa (Fig. 1). The phylogenetic analysis of NHEs possessing a CNBD among representative species of eukaryotes shows that sNHE forms an isolated group in Metazoa separated from the other NHEs in non-metazoan species (Additional file 4 Figure S1). For example, an NHE closely related to sNHE of *Arabidopsis thaliana* named SOS1 [21] possesses a CNBD, but not a VSD (Additional file 4 Figure S1); we thus considered this to be a homologue of sNHE. This result suggests that sNHE that acquired a VSD might have emerged early in the evolution of the Metazoa. However, we cannot rule out the possibility that non-metazoan organisms that conserve sNHE exist but have not been found yet−or used to exist but are now extinct.

On the other hand, sAC can be found across the eukaryotes (Fig. 1), as reported previously [27, 32, 48, 49]. The phylogenetic analysis of all ACs without transmembrane segments shows that this enzyme is widely distributed in eukaryotes, but does not form an isolated group within them (Additional file 5 Figure S2). This heterologous distribution may be attributable to our definition of sAC. In other words, we excluded ACs that lack the P-loop NTPase domain, even if some of them might have once possessed this domain but lost it during evolution. In fact, the P-loop NTPase domain is also found in bacterial sAC [27, 49].

**Fig. 5:**
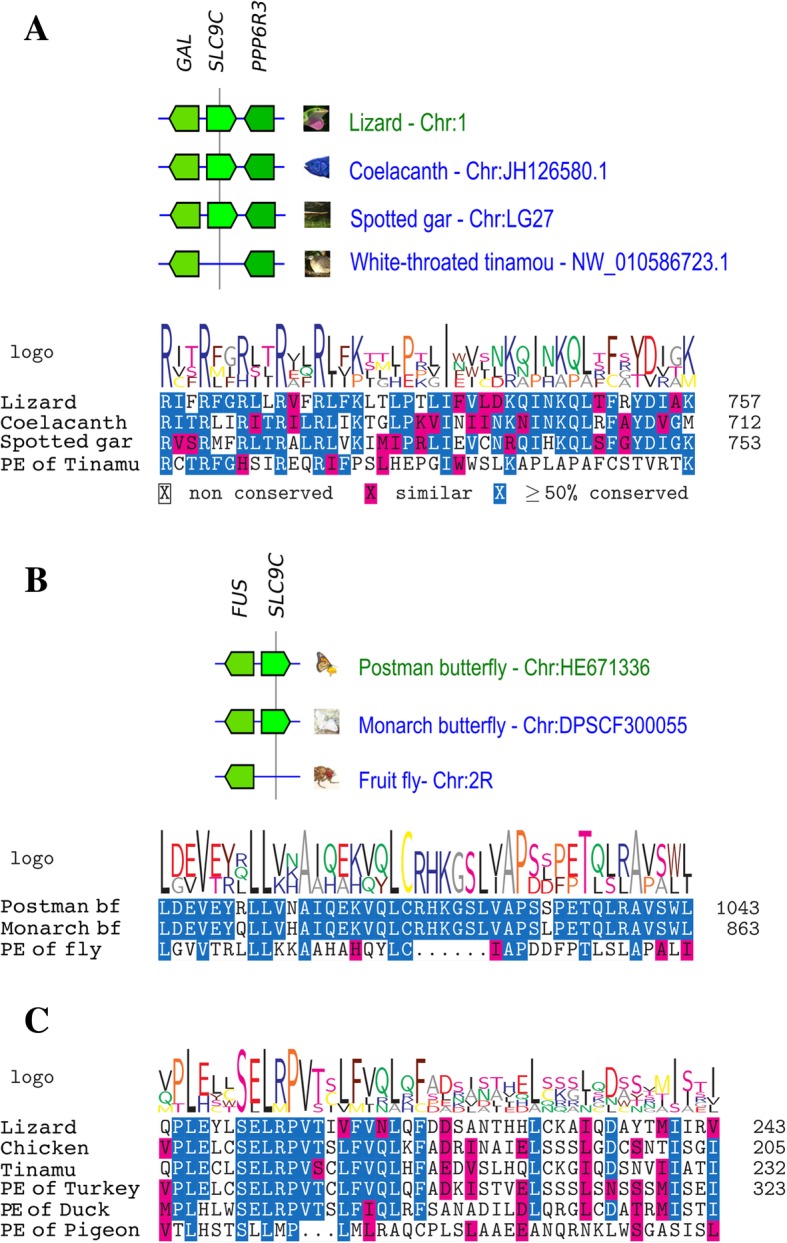
Shared synteny for *SLC9C* and pseudogenization of *SLC9C* and *ADCY10*. A. The upper panel shows synteny block comparisons around *SLC9C* among lizard, coelacanth, spotted gar, and white-throated tinamou. The lower panel shows an alignment of representative protein fragments of sNHE from lizard, coelacanth, spotted gar and the deduced amino acid sequence of a predicted exon obtained from white-throated tinamou (PE of Tinamu). B. The upper panel shows synteny block comparisons around *SLC9C* among postman butterfly, monarch butterfly and fruit fly. The lower panel shows an alignment of representative protein fragments of sNHE from the two butterflies (bf) and the deduced amino acid sequence of a predicted exon obtained from the fruit fly (PE of Fly). C. An alignment of representative protein fragments of sAC from lizard, chicken and white-throated tinamou (Tinamu) and the deduced amino acid sequences of three PE obtained from the turkey, duck, and pigeon. In synteny blocks, the arrangement reflects the order and orientation of genes in each indicated chromosome. In the case of the white-throated tinamou, the name of the unplaced genomic scaffold is indicated instead of the chromosome number. Chromosomes (Chr) are not represented to the scale of base-pair length. In all alignments, coloured highlights indicate ≥50% conserved (blue) and similar (pink) amino acid. Abbreviations for orthologues are listed in Table S2

CatSper pseudogenization was reported previously by Cai and Clapham in 2008 [16], suggesting that species lacking CatSper genes at present used to possess them, but then lost them throughout evolution. Likewise, we found a pseudogene for sNHE in the genome of the bird, *Tinamus guttatus*, using shared synteny among coelacanth, lizard, and this bird (Fig. 5A). We also found a pseudogene for sNHE in the fruit fly using the shared synteny of two butterflies (Fig. 5B). These results suggest that all species lacking sNHE at present in Metazoa used to possess this protein, as with CatSper. On the other hand, we did not find any conserved synteny for sAC among different taxonomic groups in vertebrates (data not shown), which made it difficult for us to focus on this region in analysing the pseudogene. However, in some birds, we were able to identify some predicted exons that encode an uncharacterized protein similar to sAC using TBlasN. Figure 5C shows an alignment of fragments of sAC obtained from lizard, chicken, and white-throated tinamou together with tree deduced amino acid sequences obtained from the predicted exons of turkey, duck, and pigeon (indicated with asterisks in Fig. 2). This evidence supports that bird groups lacking sAC at present used to possess this protein.

In Metazoa, distributions of sNHE and sAC are highly correlated with that of CatSper (Figs. 1−4). Actually, many phylogenetic groups were classified into ‘All’ or ‘None’. We observed that most of the species of Metazoa that possess CatSper also conserve sNHE and sAC, such as mammals, reptiles, the coelacanth, cartilaginous fishes, tunicates, amphioxus, Echinodermata, Brachiopoda, Cnidaria and Ctenophora (Fig. 1). Furthermore, we found that three species from ray-finned fishes conserving CatSper [50] also possess sNHE and sAC (Fig. 3). The shared distribution of CatSper, sNHE and sAC suggests coevolution among these three proteins in Metazoa. However, we found some exceptions to ‘All’ or ‘None’ in our classification. Two primitive birds—*Apteryx australis* and *Tinamus guttatus*—conserve CatSper and sAC, but not sNHE (Fig. 2). Likewise, we found there are several taxonomic groups that conserve sNHE and sAC without CatSper, such as Mollusca (Fig. 1) and some arthropods (Fig. 4). In addition, some species possess only sAC as some birds (Fig. 2), some arthropods (Fig. 4) and Placozoa (Fig. 1).

The presence of intermediate groups opens up the question of the nature of coevolution among these three proteins. Therefore, we performed a statistical analysis for the coexistence of the three proteins using the fewest events for the loss of a gene (or genes) based on our results (Additional file 7 Figure S4). We determined the correlation coefficient for each pair of the three proteins using the binary codes derived from Additional file 7 Figure S4 (Additional file 8 Figure S5A). As a negative control, we also determined the correlation coefficient using a randomized variable. Moderate positive correlations for each pair among the three proteins (Additional file 8 Figure S5B) indicates that the presence of intermediate groups does not contraindicate the idea of coevolution among them.

### Distribution of the NHAs (SLC9B family)

**Fig. 6:**
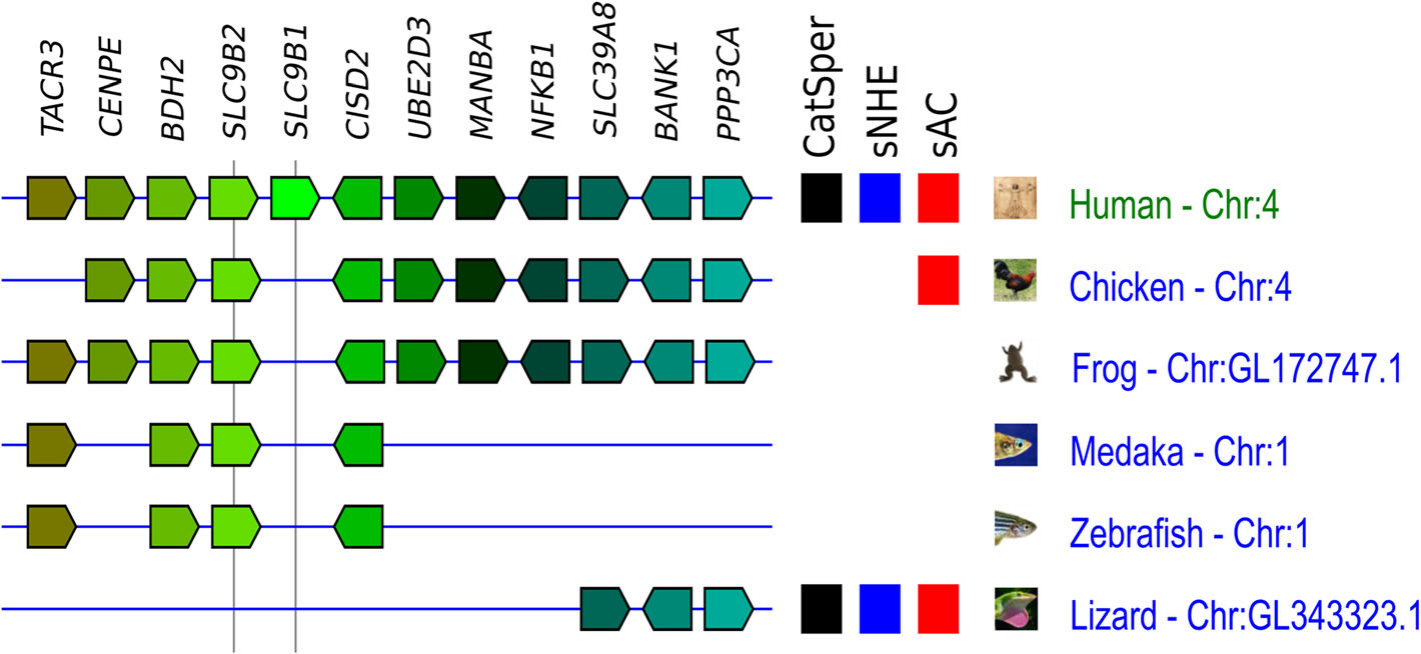
Synteny block comparison of *SLC9B* (NHA) and distribution of genes encoding CatSper, sNHE and sAC in vertebrates. Synteny blocks around *SLC9B1* (NHA1) and *SLC9B2* (NHA2) among human, chicken, frog, medaka, zebrafish, and lizard are shown together with distributions of genes encoding CatSper (black), sNHE (blue) and sAC (red). In synteny blocks, the arrangement reflects the order and orientation of genes in each chromosome. Chromosomes (Chr) are not represented to the scale of base-pair length

NHA1 (SLC9B1) was reported as a testis-specific NHE in mammals [51–53]. The importance of NHA1 and NHA2 (SLC9B2) was demonstrated by genetically modified animals; single knock-out (NHA1 KO) mice show a slight reduction in male fertility, and double knock-out (NHA1/2 dKO) mice show complete male infertility [54]. Because NHA1 and NHA2 share some properties with sNHE, such as localization at the principal piece of the flagellum and a phenotype of the null mutant (defective sperm motility and a decrease in sAC expression), we evaluated the distribution of NHAs in vertebrates. A simple search in Genomicus v. 91.01 revealed that mammalian NHA1 and NHA2 are tandemly localized in chromosome 4 in most mammals, including humans (Fig. 6), indicating that one of these genes is the product of gene duplication. The same search showed that *SLC9B* can be found in most vertebrates, including turkey and chicken, in which only sAC is conserved, and in the frog, medaka and zebrafish, in which none of the three proteins (CatSper, sNHE and sAC) is conserved. Conversely, lizards—conserving all three proteins—do not show conservation of NHA. These results indicate that NHAs do not apparently correlate with the conservation of CatSper, sNHE, or sAC.

## Discussion

### Coevolution of CatSper, sNHE and sAC in Metazoa

Here, we performed a comparative genomic analysis of CatSper, sNHE and sAC throughout the eukaryotes and found a unique and restricted distribution pattern of each protein. In Metazoa, the distribution of CatSper, sNHE and sAC shows an apparent pattern of coevolution among the three proteins, suggesting that sNHE and sAC are key regulators of CatSper in this taxon. Notably, similar gene distribution patterns are reported for the mitochondrial Ca^2+^ channel complex composed of MCU/MCUb, MICU1/MICU2, and EMRE [55, 56]. For example, *Saccharomyces cerevisiae* lacks MCU, and this species does not conserve either MICU or EMRE [56].

It has been revealed that CatSper is up-regulated by pH_c_ alkalization in mammals and sea urchins [3, 6, 7, 19] and the mechanism of sNHE activation through VSD and CNBD has been confirmed in the sea urchin [26]. Considering the coexistence of sNHE to CatSper in Metazoa (Figs. 1 and 3) with minimum exceptions among Mollusca (Fig. 1), primitive birds (Fig. 2), and arthropods (Fig. 3), sNHE must be a general regulator (activator) of CatSper in the Metazoa. Because extracellular Na^+^ is an essential factor for any Na^+^/H^+^ exchangers to increase pH_c_, the activity of sNHE should be altered for external fertilizers living in freshwater (< 10 mM Na^+^). This may explain why amphibians and most ray-finned fishes living in freshwater do not conserve sNHE (Figs. 1 and 3). However, the three ray-finned fishes in which sNHE is conserved reproduce in freshwater. This apparent discrepancy in the function of sNHE in these species remains a question to be resolved.

The activity of sea urchin sNHE is up-regulated by cAMP binding to the CNBD [26]. Our findings imply that sNHE and sAC work together as important regulators of CatSper in general. On the other hand, it was demonstrated recently that cAMP positively regulates CatSper through protein kinase A (PKA) in mouse spermatozoa [20]. This report suggests that cAMP can modulate CatSper independently of sNHE, which may explain the case of primitive birds (or ray-finned fishes) in which CatSper and sAC are conserved, but which lack sNHE (or lack its activity in freshwater). In Metazoa, species in which CatSper is conserved also without exception show conservation of sAC (Fig. 1). Our results support the importance of cAMP for CatSper activation.

In the fruit fly *D. melanogaster*, none of the three proteins are conserved in its genome (Fig. 4) [16]. Instead, it is known in that the transient receptor potential polycystic (TRPP)2/polycystic kidney disease (PKD)2 channel is essential for sperm flagellar beat regulation [57, 58]. It has been reported that the genes encoding PKD2 channels in fruit flies have notable diversity (indicating rapid evolution) between two closely related species compared with other housekeeping genes [59]. This observation is a typical feature of proteins involved in sexual reproduction [60, 61] including CatSper [59, 62, 63]. Because Ca^2+^ plays a fundamental role in flagellar beat regulation, species that lack CatSper should have their own substitute for CatSper. It would be interesting to explore whether all such substitutes in different taxonomic groups show the same property of positive selection for CatSper in mammals and TRPP2 in fruit flies.

### VSD of sNHE

A notable structural feature of sNHE compared with other NHEs is that this exchanger possesses two predicted domains: VSD and CNBD. Almost all of the sNHEs we analysed show conservation of the four predicted transmembrane segments (S1-S4) and the key amino acids in each segment corresponding to known VSDs [64, 65] (Additional file 6: Figure S3). However, in the case of the sea walnut (ctenophore) exchanger, we observed a markedly degraded VSD (only S1 is conserved), suggesting that the voltage sensitivity of this sNHE is abolished.

In non-metazoan species, we did not find any exchangers with a predicted VSD. Instead, we found some NHEs with a predicted CNBD, such as SOS1 found in *Arabidopsis thaliana*. However, SOS1 is expressed in somatic cells rather than germinal cells [66, 67]. This exchanger may thus not be involved in sexual reproduction. Actually, CatSper and sNHE can be found at least in the species that conserve an axoneme (i.e. *C. elegans* does not conserve either of these proteins) (Additional file 9 Figure S6). This fact supports the idea that the principal role of CatSper is to regulate the shape of the flagellar or ciliary beat of the male gamete. Therefore, we consider that SOS1 is a homologue of sNHE rather than an orthologue, because *A. thaliana* has not conserved the axoneme [68].

In the 1980s, Lee intensively studied the presence and function of an unusual NHE that is activated by Vm hyperpolarization [69–71]. Ever since the first primary structure of sNHE containing a putative VSD was reported in the mouse [21], the unusual regulation of the NHE in sea urchin spermatozoa has been attributed to this putative VSD of sNHE [72–74]. The activities of VSD and CNBD of sNHE in sea urchin spermatozoa have now been confirmed by expression of the recombinant sNHE and its site-directed mutagenesis [26]. Although a successful expression of mouse sNHE has not yet been reported, there is a report that suggests the activation of sNHE by Vm hyperpolarization in mouse spermatozoa [75]. Therefore, sNHE may be up-regulated by Vm hyperpolarization in general. However, some sNHEs, such as human sNHE, could be independent of Vm [26, 76]. In the sea urchin, the tetrameric potassium-selective cyclic nucleotide gated (tetraKCNG/CNGK) channel is known to be responsible for the initial Vm hyperpolarization in chemoattractant signal transduction [77–79], which leads to the pH_c_ increase mediated by sNHE [80–82], and repolarization occurs through hyperpolarization-activated and cyclic nucleotide-gated (HCN) channels [83]. Subsequently, cytoplasmic Ca^2+^ concentration rises due to Ca^2+^ influx through CatSper [19, 84]. Sperm chemotaxis has been observed in distinct taxonomic groups of marine invertebrates such as tunicates, Echinodermata, Mollusca and Cnidaria [85] and most of them conserve tetraKCNG/CNGK [86]. Interestingly, these taxonomic groups also conserve CatSper, sNHE and sAC (Fig. 1). It will be important to explore whether the signalling cascade of the chemoattractant established in sea urchin sperm is conserved in other marine invertebrates. On the other hand, in many vertebrates, including mammals, Slo3 appears to have replaced the function of tetraKCNG/CNGK [87]. Namely, in the mouse, Slo3 together with sNHE and sAC seem to play an important role in controlling CatSper.

### The significance of intermediates between ‘all’ and ‘none’

Although the ‘All’ and ‘None’ categories of the three proteins were two major patterns we found in Metazoa, there are a few intermediate groups. As two notable examples, we found species lacking CatSper in which sNHE and sAC (‘Int1’) are conserved, and species in which only sAC (‘Int2’) is conserved. Furthermore, we found exceptional cases of only two species of birds in which CatSper and sAC, but not sNHE, are conserved (defined as ‘Int3’).

The group Int1 includes some arthropods (Fig. 4) and Mollusca (Fig. 1). Using the silkworm database (SilkDB described in Additional file 1: Table S1) (http://silkworm.genomics.org.cn/silkdb), we confirmed that sNHE and sAC are expressed in the testis (microarray sw10076, sw11099 and sw13918). Interestingly, there is a butterfly (*Heliconius melpomene*) that conserves two CatSper pore-forming subunits (α subunits) instead of the four subunits, and it was classified as Int1 (lacking CatSper) following the definitions used in this study. We do not know why some α subunits remain intact in these species. It would be interesting to know whether functional channels are formed by homo-tetramers or tetramers composed of two dimers in this butterfly. In addition, a similar case was observed in a bird, *Struthio camelus* (i.e. three α subunits remain intact in this species). In the Int1 group, sNHE and sAC have been preserved even after CatSper was lost. It is possible that Ca^2+^ channels that substitute for CatSper are up-regulated by pH_c_ and/or cAMP. Further studies are required to address this question.

On the other hand, the group Int2, in which only sAC is conserved, includes some birds (Fig. 2), some arthropods (Fig. 4), and *Trichoplax adhaerens* (Fig. 1; indicated as Placozoa). Although CatSper and sNHE have no reported functions in somatic cells, it has been proposed that sAC plays a role as a CO_2_/HCO_3_^−^ sensor in somatic cells [88, 89]. It has been suggested that sAC plays two roles in the Int2 group: i) modulating the activity of a Ca^2+^ channel that is a substitute for CatSper in spermatozoa and/or ii) functioning as a CO_2_/HCO_3_^−^ sensor in somatic cells.

Finally, the Int3 groups in which CatSper and sAC, but not sNHE, is conserved include only two species of primitive birds. Considering that most of the species in which CatSper is conserved also retain sNHE in Metazoa, a different proton transporter may have substituted for sNHE to alkalinize pH_c_ and control CatSper. Alternatively, CatSper in these species may have acquired a different mode of regulation. In any case, it will be instructive to study the regulation of CatSper in the Int3 group.

In the present study, we obtained moderate—but not high—positive correlations for each pair among the three proteins (Additional file 8: Figure S5B). This result probably reflects the replacement of CatSper or sNHE by another protein, and/or acquisition of a new function of sAC in some species. The reason needs to be addressed by further studies.

### Regulation of CatSper in non-metazoans

Although CatSper is supposed to have emerged at an early stage of eukaryote evolution [14], nothing is known about the regulation of CatSper in non-metazoans, including the question of pH_c_ dependence. At least in the Metazoa, CatSper is probably up-regulated by pH_c_ alkalization principally through sNHE. However, considering that sNHE is found only in Metazoa, CatSper in non-metazoan eukaryotes may be regulated by other proteins that control pH_c_, as in human spermatozoa [90]. Alternatively, CatSper in non-metazoan species may not be strongly regulated by pH_c_. On the other hand, sAC could be involved in CatSper regulation in non-metazoans as this is protein widely distributed in both prokaryotes and eukaryotes, and most of the non-metazoan species that possess CatSper also preserve sAC. Therefore, it is feasible that activation of CatSper by cAMP, probably through PKA [20], is a more primitive mechanism to regulate CatSper, rather than pH_c_. Studies of CatSper in non-metazoan species will be essential to address these issues.

## Conclusions

Differences within closely related clades and commonalities between distant species are unique and notable features of the regulatory mechanisms of sperm motility, which are interesting, but sometimes troublesome, for gaining a better understanding of the mechanism in a particular species [50, 91, 92]. As a prominent case, loss of CatSper may have occurred independently in different taxonomic groups of eukaryotes at distinct time points, leading to the current heterogeneous distributions of this channel in all eukaryotes [14, 16, 17]. Our comparative genomic analysis revealed coevolutionary linkages among CatSper, sNHE and sAC in the Metazoa, suggesting that these three proteins form prototypical machinery to regulate animal sperm flagellar beating. Loss of any of the three proteins might be attributed to the acquisition of alternative component(s) in the sperm signalling pathways by chance, or by adaptation of a new fertilization environment, which may have evolved independently in distinct taxonomic groups. In addition, our study suggests that the regulation of CatSper in non-metazoan is different from that of Metazoa. Further experiments are required to address the many questions raised in this study to understand the regulation of sperm motility in diverse species.

## Additional files

Additional file 1: **Table S1.** Databases used for determination of the presence of CatSper, sNHE and sAC. (PDF 2174 kb)

Additional file 2: **Table S2.** Abbreviations for orthologues used in Fig. 5. (PDF 2440 kb)

Additional file 3: **Table S3.** Sequeces of sNHE and sAC used for Molecular Phylogenetic analyses (PDF 3728 kb)

Additional file 4: **Figure S1.** Molecular phylogeny and domain compositions of sNHE and its homologues. (PDF 312 kb)

Additional file 5: **Figure S2.** Molecular phylogeny and domain compositions of sAC and its homologues (PDF 68 kb)

Additional file 6: **Figure S3.** Representative alignment for the four transmembrane segments of the VSD (PDF 917 kb)

Additional file 7: **Figure S4.** The least gene-loss events of CatSper, sNHE and sAC in the Metazoa. (PDF 55 kb)

Additional file 8: **Figure S5.** Statistical analysis of the coexistence of CatSper, sNHE and sAC. (PDF 62 kb)

Additional file 9: **Figure S6.** Distribution of genes encoding the three proteins in representative species with and without an axoneme. (PDF 98 kb)

## Data Availability

The datasets supporting the conclusions of this article are included within the article.
