# Supplementary material for: Comparative genomic analysis suggests that the sperm-specific sodium/proton exchanger and soluble adenylyl cyclase are key regulators of CatSper among the Metazoa
Source: Zoological Lett. 2019 Jul 26;5:25. doi: 10.1186/s40851-019-0141-3 (PMC6660944; doi:10.1186/s40851-019-0141-3)
Supplement: Supplementary file 1 — Table S1. Databases used for determination of the presence of CatSper, sNHE and sAC. (PDF 2174 kb) [file 40851_2019_141_MOESM1_ESM.pdf]

Fig. S1

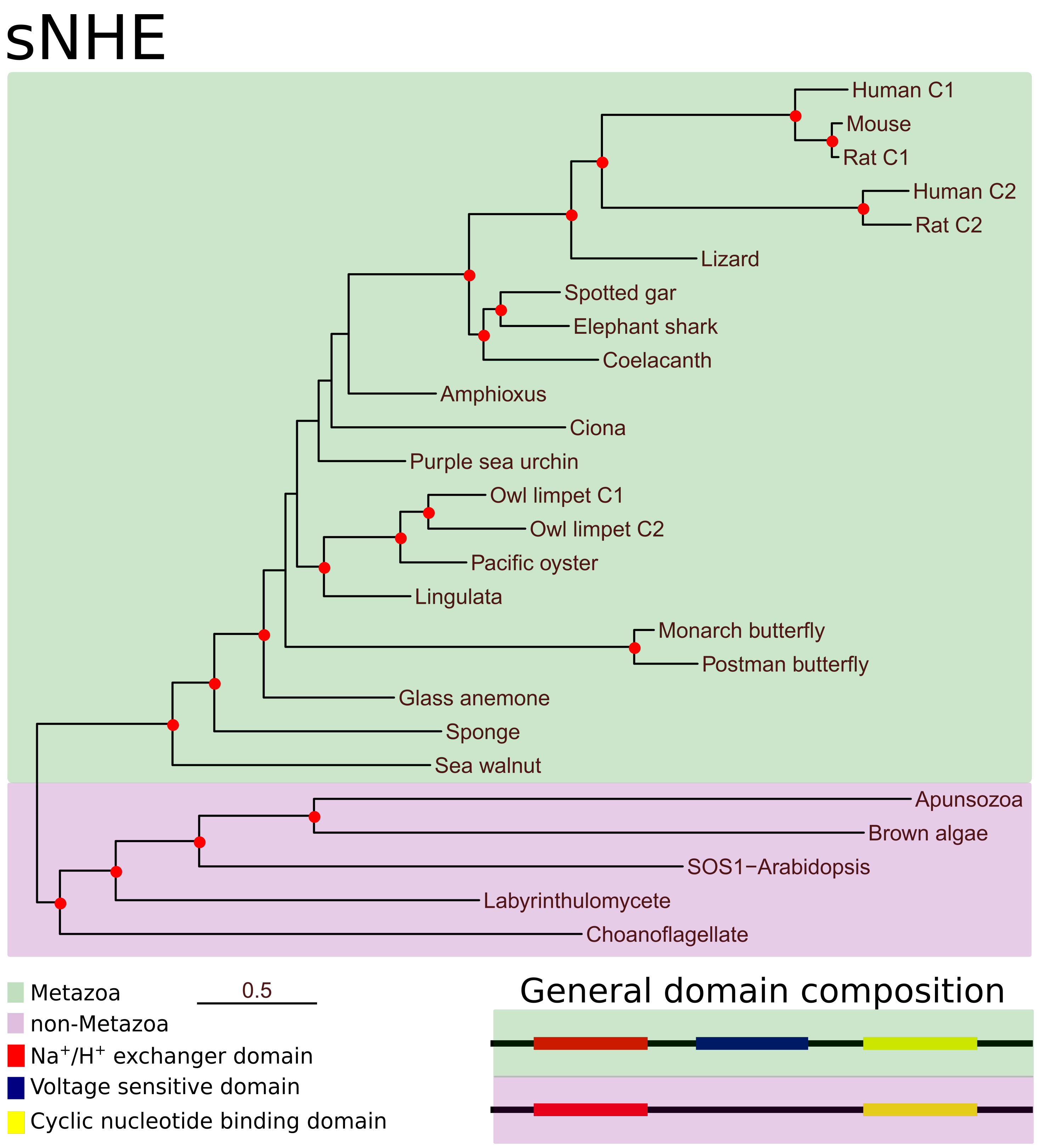

Figure S1. Molecular phylogeny and domain compositions of sNHE and its homologues. The evolutionary history was inferred among orthologues and homologues of sNHE. Metazoan sNHE sequences clustered together (green) while non-metazoan sNHE species clustered apart (purple). General domain compositions for sNHE (green) and sNHE homologue (purple) are represented as the Na^+/H^+ exchanger domain (red box), voltage-sensor domain (blue box) and cyclic nucleotide-binding domain (yellow box). The tree obtained by the highest log likelihood is shown with branch lengths measured in the number of substitutions per site. Red dots next to the branches indicate that the bootstrapping values are higher than 50%. A discrete Gamma distribution was used to model evolutionary rate differences among sites (two categories (+G, parameter = 1.73)).
