# Supplementary material for: Comparative genomic analysis suggests that the sperm-specific sodium/proton exchanger and soluble adenylyl cyclase are key regulators of CatSper among the Metazoa
Source: Zoological Lett. 2019 Jul 26;5:25. doi: 10.1186/s40851-019-0141-3 (PMC6660944; doi:10.1186/s40851-019-0141-3)
Supplement: Supplementary file 2 — Table S2. Abbreviations for orthologues used in Fig. 5. (PDF 2440 kb) [file 40851_2019_141_MOESM2_ESM.pdf]

Fig. S2

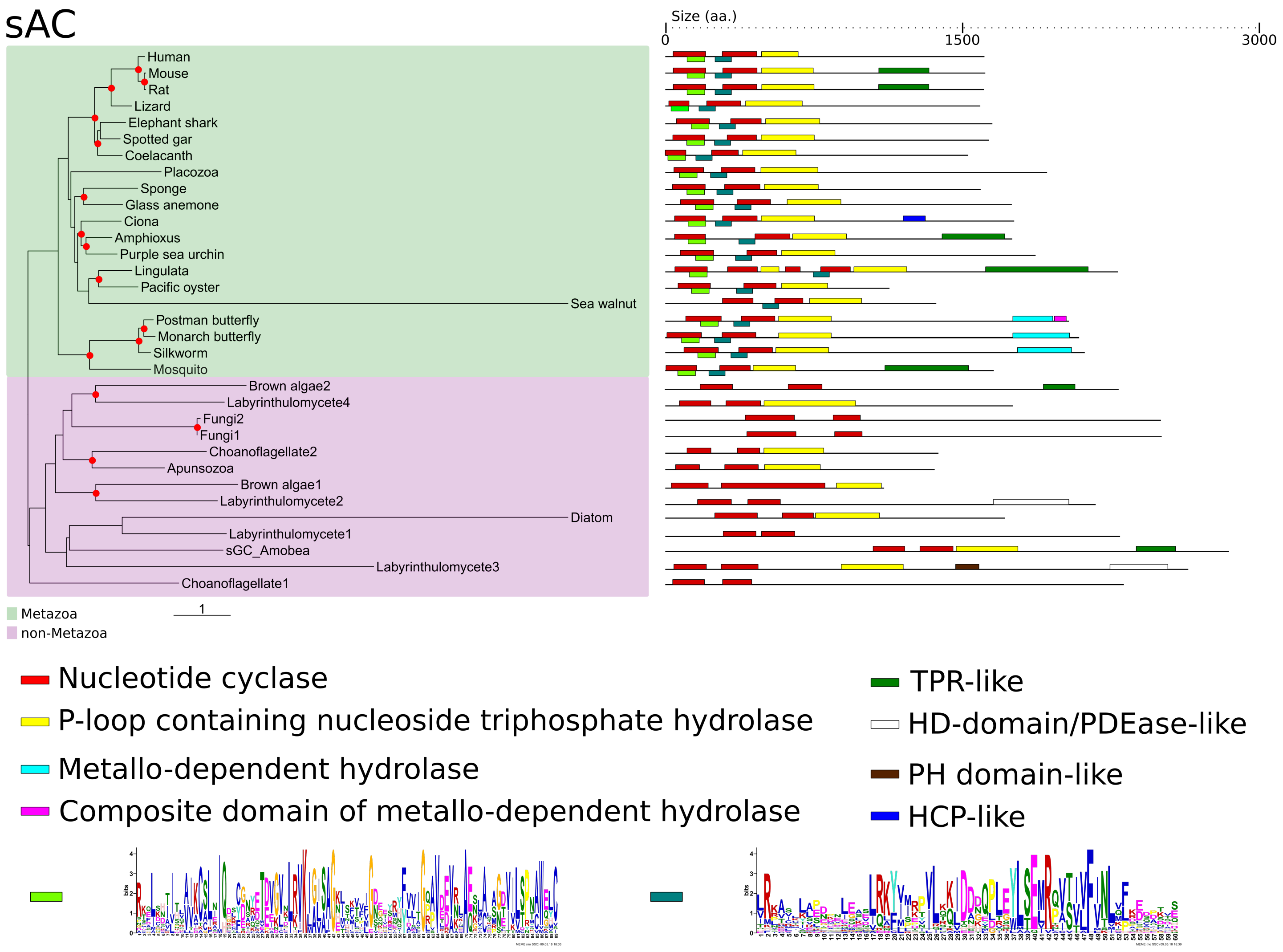

Figure S2. Molecular phylogeny and domain compositions of sAC and its homologues. The evolutionary history was inferred among orthologs and homologues of sAC. Metazoan sAC sequences clustered together (green) while sAC and its homologs were distributed heterogeneously (purple). According to the results obtained from SUPERFAMILY analysis, we observed several diversifications of the C-terminal of sAC, the domain compositions are described as follows: nucleotide cyclase domain (red box), P-loop containing nucleoside triphosphate hydrolase (P-loop NTPase, yellow box), metallo-dependent hydrolase domain (cyan box), composite domain of metallo-dependent hydrolase (pink box), TPR-like domain (dark green box), HD-domain/PDEase-like domain (white box), PH-like domain (brown box) and HCP-like domain (blue box). Furthermore, the MEME analysis performed showed two Metazoa-specific motifs (light green and dark cyan boxes below catalytic domains). The tree obtained by the highest log likelihood is shown with branch lengths measured from the number of substitutions per site. Red dots next to the branches indicate that the bootstrapping values are >50 %. A discrete Gamma distribution was used to model evolutionary rate differences among sites (two categories (+G, parameter = 1.66)).
