# Supplementary material for: Comparative genomic analysis suggests that the sperm-specific sodium/proton exchanger and soluble adenylyl cyclase are key regulators of CatSper among the Metazoa
Source: Zoological Lett. 2019 Jul 26;5:25. doi: 10.1186/s40851-019-0141-3 (PMC6660944; doi:10.1186/s40851-019-0141-3)
Supplement: Supplementary file 3 — Table S3. Sequeces of sNHE and sAC used for Molecular Phylogenetic analyses (PDF 3728 kb) [file 40851_2019_141_MOESM3_ESM.pdf]

Fig. S3

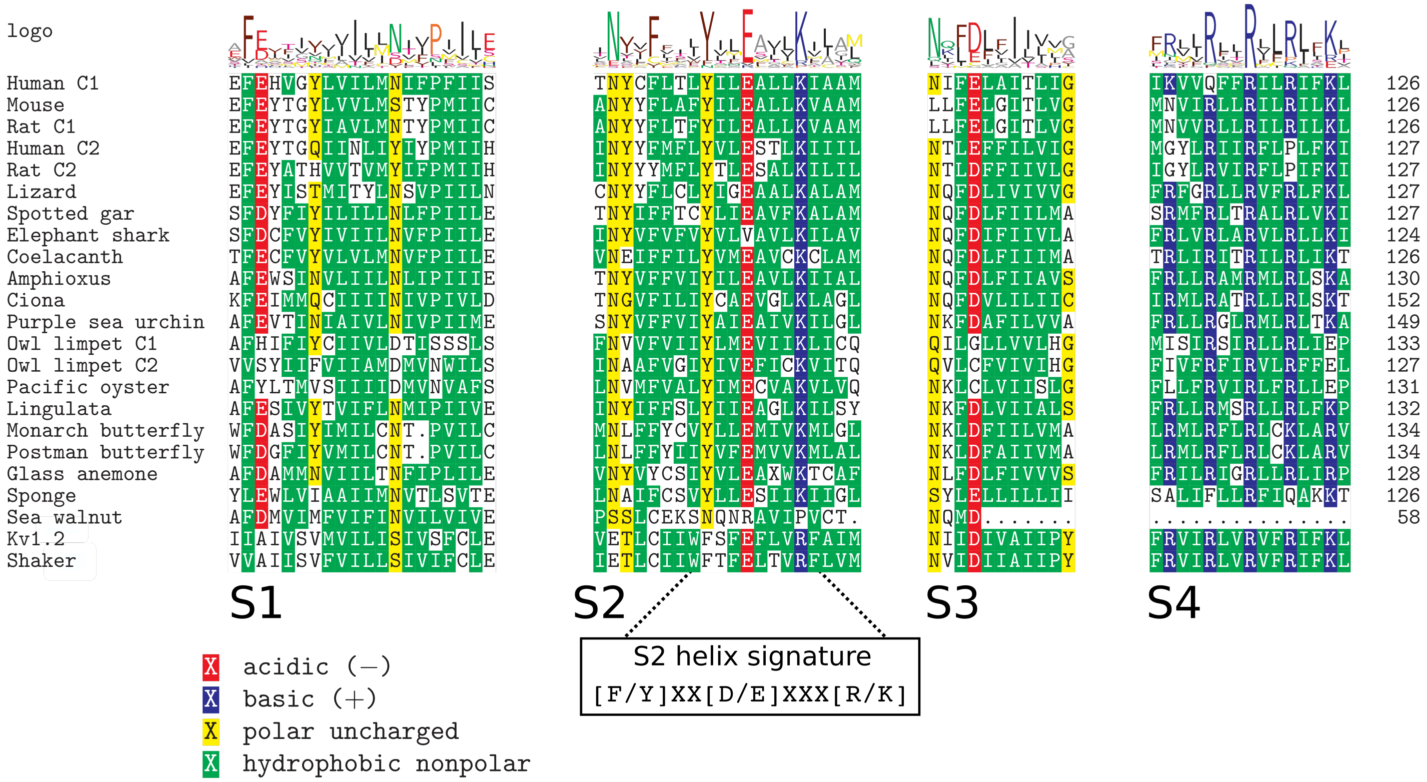

Figure S3. Representative alignment for the four transmembrane segments of the VSD. Transmembrane segments (S1−S4) of sNHE for at least one representative species from each taxonomic group were analysed. We added two functional VSDs of Kv1.2 and Shaker into the alignment. Conserved amino acids are highlighted according to their chemical properties: negatively charged (red), positively charged (blue), polar uncharged (yellow) and hydrophobic nonpolar (green). The conserved S2 helix signature [F/Y]XX[D/E]XXX[R/K] is shown below the alignment, where X represents any amino acid.
