# Supplementary material for: Comparative genomic analysis suggests that the sperm-specific sodium/proton exchanger and soluble adenylyl cyclase are key regulators of CatSper among the Metazoa
Source: Zoological Lett. 2019 Jul 26;5:25. doi: 10.1186/s40851-019-0141-3 (PMC6660944; doi:10.1186/s40851-019-0141-3)
Supplement: Supplementary file 4 — Figure S1. Molecular phylogeny and domain compositions of sNHE and its homologues. (PDF 312 kb) [file 40851_2019_141_MOESM4_ESM.pdf]

Fig. S4

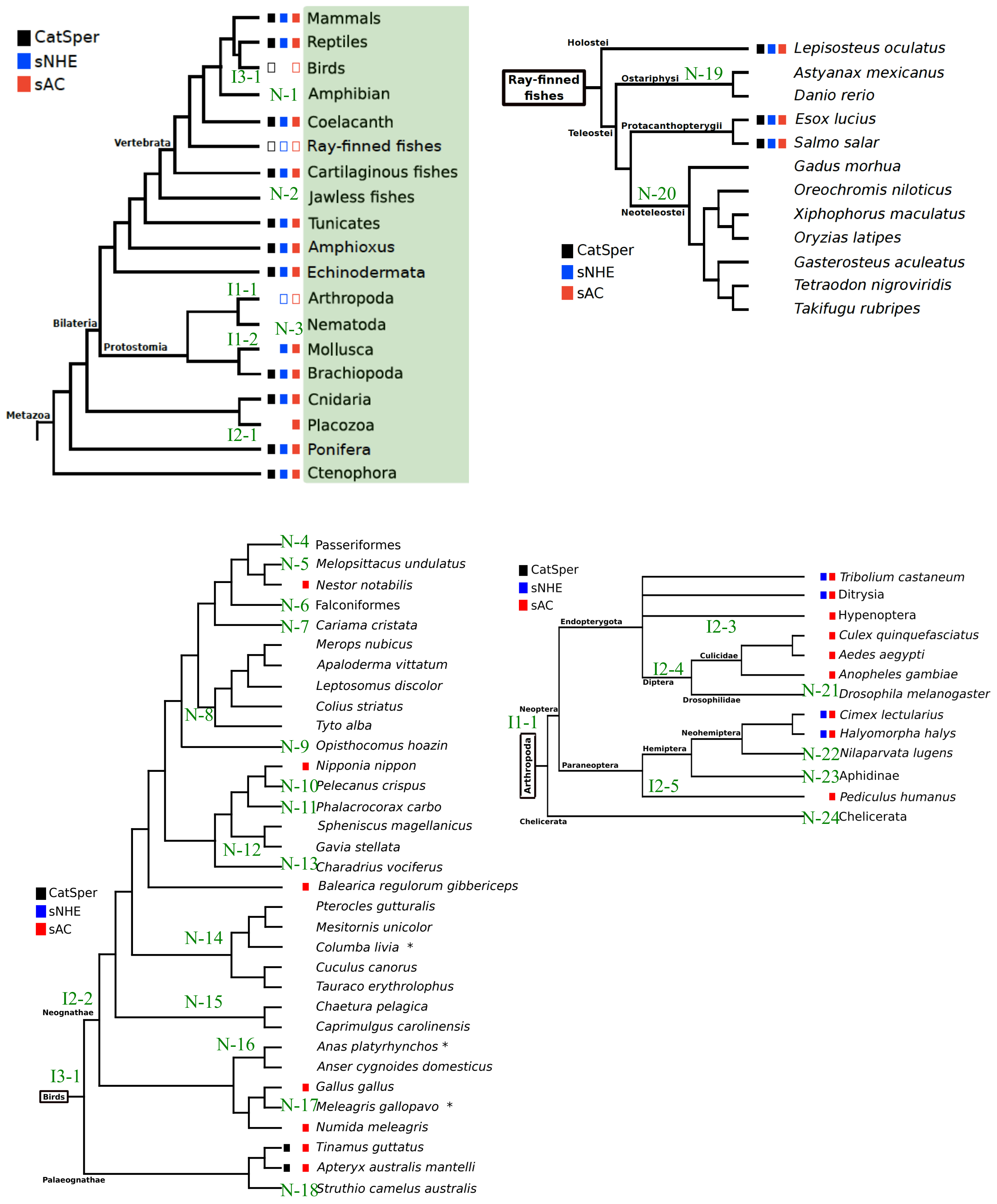

Figure S4. The least gene-loss events of CatSper, sNHE and sAC in the Metazoa.
The putative (fewest possible) events for the loss of gene(s) are described on the same phylogenetic trees used in Figs1−4 with green letters. Symbols I1, I2, I3 and N represent gene-loss patterns of Int1 (loss of CatSper), Int2 (loss of CatSper and sNHE), Int3 (loss of sNHE) and None (loss of all three proteins), respectively. A number followed by each symbol represents an independent gene-loss event but does not represent the time sequence.
