# Supplementary material for: Comparative genomic analysis suggests that the sperm-specific sodium/proton exchanger and soluble adenylyl cyclase are key regulators of CatSper among the Metazoa
Source: Zoological Lett. 2019 Jul 26;5:25. doi: 10.1186/s40851-019-0141-3 (PMC6660944; doi:10.1186/s40851-019-0141-3)
Supplement: Supplementary file 5 — Figure S2. Molecular phylogeny and domain compositions of sAC and its homologues (PDF 68 kb) [file 40851_2019_141_MOESM5_ESM.pdf]

Fig. S6

A

#	Case	CatSper	sNHE	sAC
1	I1-1	0	1	1
2	I1-2	0	1	1
3	I2-1	0	0	1
4	I2-2	0	0	1
5	I2-3	0	0	1
6	I2-4	0	0	1
7	I2-5	0	0	1
8	I3-1	1	0	1
9	N-1	0	0	0
10	N-2	0	0	0
11	N-3	0	0	0
12	N-4	0	0	0
13	N-5	0	0	0
14	N-6	0	0	0
15	N-7	0	0	0
16	N-8	0	0	0
17	N-9	0	0	0
18	N-10	0	0	0
19	N-11	0	0	0
20	N-12	0	0	0
21	N-13	0	0	0
22	N-14	0	0	0
23	N-15	0	0	0
24	N-16	0	0	0
25	N-17	0	0	0
26	N-18	0	0	0
27	N-19	0	0	0
28	N-20	0	0	0
29	N-21	0	0	0
30	N-22	0	0	0
31	N-23	0	0	0
32	N-24	0	0	0
33	A	1	1	1

B

Correlation coefficient *r*
(p-Value of coefficient)

	CatSper	sNHE	sAC
sNHE	0.36<br>(0.04)		
sAC	0.42<br>(0.02)	0.52<br>(0.002)	
X	-3.4×10^{-5}<br>(0.5)	-3.4×10^{-5}<br>(0.5)	8.2×10^{-5}<br>(0.5)

Figure S5. Statistical analysis of the coexistence of CatSper, sNHE and sAC
A. The binary code table represents existence (1) or absence (0) of the three genes in each gene-loss event we propose including a single A (group 'All') that conserves all three genes. B. The correlation coefficient *r* of each pair is shown together with its *P* value in parenthesis. Symbol X indicates a randomized variable carried out 500,000 times for each protein.
