# Supplementary material for: Comparative genomic analysis suggests that the sperm-specific sodium/proton exchanger and soluble adenylyl cyclase are key regulators of CatSper among the Metazoa
Source: Zoological Lett. 2019 Jul 26;5:25. doi: 10.1186/s40851-019-0141-3 (PMC6660944; doi:10.1186/s40851-019-0141-3)
Supplement: Supplementary file 6 — Figure S3. Representative alignment for the four transmembrane segments of the VSD (PDF 917 kb) [file 40851_2019_141_MOESM6_ESM.pdf]

Fig. S6

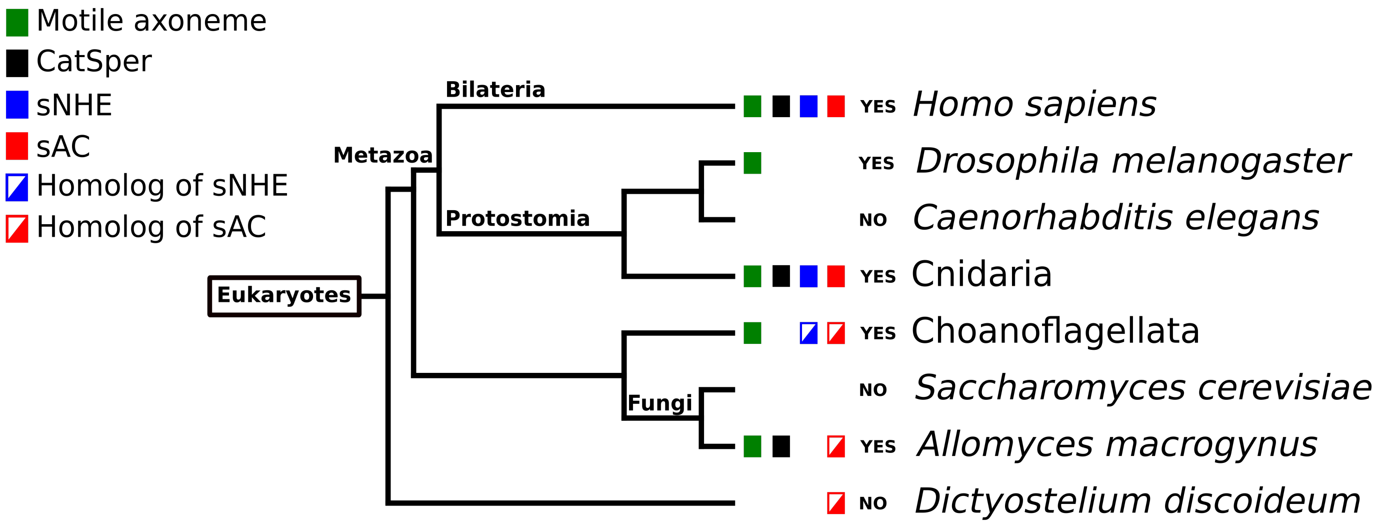

Figure S6. Distribution of genes encoding the three proteins in representative species with and without an axoneme. Boxes represent the presence of genes as shown in Figure 1 with additional green box for species conserving the motile axoneme. The phylogenetic tree was prepared based on the Tree of Life project (http://www.tolweb.org/tree/). The branching patterns do not represent a proportional evolutionary rate.
