# Supplementary material for: Comparative genomic analysis suggests that the sperm-specific sodium/proton exchanger and soluble adenylyl cyclase are key regulators of CatSper among the Metazoa
Source: Zoological Lett. 2019 Jul 26;5:25. doi: 10.1186/s40851-019-0141-3 (PMC6660944; doi:10.1186/s40851-019-0141-3)
Supplement: Supplementary file 7 — Figure S4. The least gene-loss events of CatSper, sNHE and sAC in the Metazoa. (PDF 55 kb) [file 40851_2019_141_MOESM7_ESM.pdf]

Table 1. Databases used to determinate the presence of CatSper, sNHE and sAC.

Database	URL	Reference
National Center for Biotechnology Information	http://www.ncbi.nlm.nih.gov/	(S. Altschul et al. 1997)
STRING 10	http://string-db.org/	(Franceschini et al. 2013)
AGRIPEST BASE	http://agripestbase.org/	
InsectBase	http://www.insect-genome.com/	(Yin et al. 2015)
e!Ensembl	http://www.ensembl.org/	(Kersey et al. 2015)
e!EnsemblMetazoa	http://Metazoa.ensembl.org/	(Kersey et al. 2015)
e!EnsemblFungi	http://fungi.ensembl.org/	(Kersey et al. 2015)
e!EnsemblProtist	http://protists.ensembl.org/	(Kersey et al. 2015)
Broad Institute / Origins of Multicellularity	http://www.broadinstitute.org/	
SkateBase	http://skatebase.org/	(Wyffels et al. 2014;Wang et al. 2012)
Genome Portal	http://genome.jgi.doe.gov/	(Nordberg et al. 2014)
Phytozome	https://phytozome.jgi.doe.gov/	(Nordberg et al. 2014)
Metazome	https://metazome.jgi.doe.gov	(Nordberg et al. 2014)
SilkDB	http://silkworm.genomics.org.cn/	(J. Wang et al. 2005; Xia et al. 2004)
FlyBase	http://flybase.org/	(Attrill et al. 2016)
