# Supplementary material for: Comparative genomic analysis suggests that the sperm-specific sodium/proton exchanger and soluble adenylyl cyclase are key regulators of CatSper among the Metazoa
Source: Zoological Lett. 2019 Jul 26;5:25. doi: 10.1186/s40851-019-0141-3 (PMC6660944; doi:10.1186/s40851-019-0141-3)
Supplement: Supplementary file 8 — Figure S5. Statistical analysis of the coexistence of CatSper, sNHE and sAC. (PDF 62 kb) [file 40851_2019_141_MOESM8_ESM.pdf]

Table S2 Abbreviations for orthologues for figure 5

Abbreviation	Species	Ensembl or NCBI Identification	Description
**Figure 5A**			
**Abbreviation**	**Species**	**Ensembl or NCBI Identification**	**Description**
GAL	Lizard	ENSACAT00000014193	Galanin and GMAP prepropeptide
SLC9C	Lizard	ENSACAT00000014259	Na+/H+ exchanger
PPP6R3	Lizard	ENSACAT0000001440	Protein phosphatase 6 regulatory subunit 3
GAL	Coelacanth	ENSLACG00000018714	Galanin and GMAP prepropeptide
SLC9C	Coelacanth	ENSLACG00000018675	Na+/H+ exchanger
PPP6R3	Coelacanth	ENSLACG00000018653	Protein phosphatase 6 regulatory subunit 3
GAL	Spotted gar	ENSLOCG00000002486	Galanin and GMAP prepropeptide
SLC9C	Spotted gar	ENSLOCG00000002512	Na+/H+ exchanger
PPP6R3	Spotted gar	ENSLOCG00000002546	Protein phosphatase 6 regulatory subunit 3
GAL	Tinamu	XP_010220310.1	Galanin and GMAP prepropeptide
PPP6R3	Tinamu	XP_010220312.1	Protein phosphatase 6 regulatory subunit 3
**Figure 5B**			
**Abbreviation**	**Species**	**Ensembl or NCBI Identification**	**Description**
FUS	Postman butterfly	HMEL013208	FUS RNA binding protein
SLC9C	Postman butterfly	HMEL013207	Na+/H+ exchanger
FUS	Monarch butterfly	DPOGS203545	FUS RNA binding protein
SLC9C	Monarch butterfly	DPOGS203525	Na+/H+ exchanger
FUS	Fruit fly	FBgn0023441	FUS RNA binding protein
