# Supplementary material for: Comparative genomic analysis suggests that the sperm-specific sodium/proton exchanger and soluble adenylyl cyclase are key regulators of CatSper among the Metazoa
Source: Zoological Lett. 2019 Jul 26;5:25. doi: 10.1186/s40851-019-0141-3 (PMC6660944; doi:10.1186/s40851-019-0141-3)
Supplement: Supplementary file 9 — Figure S6. Distribution of genes encoding the three proteins in representative species with and without an axoneme. (PDF 98 kb) [file 40851_2019_141_MOESM9_ESM.pdf]

Table S3 Sequeces of sNHE and sAC used for Molecular Phylogenetic analyses

Sequences used for Figure S1 sNHE		
**Species**	**Common name**	Ensembl or NCBI Identification
Homo sapiens	Human C1	NM_183061.2
Mus musculus	Mouse	NM_198106.4
Rattus norvegicus	Rat C1	NM_001008762.2
Homo sapiens	Human C2	NM_178527.3
Rattus norvegicus	Rat C2	XM_017604643.1
Anole carolinensis	Lizard	XM_016993479.1
Lepisosteus oculatus	Spotted gar	XM_015338064.1
Callorhinchus milii	Elephant shark	XM_007887836.1
Latimeria chalumnae	Coelacanth	XM_014494506.1
Branchiostoma floridae	Amphioxus	XM_002597519.1
Ciona intestinalis	Ciona	XM_002119928.2
Strongylocentrotus purpuratus	Purple sea urchin	NM_001098457.1
Lottia gigantea	Owl limpet C1	XM_009055560.1
Lottia gigantea	Owl limpet C2	XM_009053155.1
Crassostrea gigas	Pacific oyster	XM_020066843.1
Lingula anatina	Lingulata	XM_013565254.1
Danaus plexippus	Monarch butterfly	DPOGS203525
Heliconius melpomene	Postman butterfly	HMEL013207
Exaiptasia pallida	Glass anemone	XM_021043824.1
Amphimedon queenslandica	Sponge	XM_003384983.2
Mnemiopsis leidyi	Sea walnut	ML174765a
Salpingoeca rosetta	Apunsozoa	XM_004995518.1
Ectocarpus silliculosus	Brown algae	*Esi0050_054
Arabidopsis thaliana	SOS1-Arabidopsis	NM_126259.4
Auratiochytrium limacinum	Labyrinthulomycete	*gw1.19.482.1
Thecamona trahens	Choanoflagellate	XM_013897693.1

** from https://genome.jgi.doe.gov/*

Sequences used for Figure S2 sAC		
**Species**	**Common name**	Ensembl or NCBI Identification
Homo sapiens	Human	XM_011509760.3
Mus musculus	Mouse	NM_173029.3
Rattus norvegicus	Rat C1	NM_021684.1
Anole lizard	Lizard	XM_008119652.2
Callorhinchus milii	Elephant shark	XM_007890197.1
Lepisosteus oculatus	Spotted gar	XM_015359032.1
Latimeria chalumnae	Coelacanth	XM_014493498.1
Trichoplax adhaerens	Placozoa	XM_002117821.1
Amphimedon queenslandica	Sponge	XM_019993565.1
Exaiptasia pallida	Glass anemone	KY853034.1
Ciona intestinalis	Ciona	XM_002121860.4
Branchiostoma floridae	Amphioxus	XM_002610098.1
Strongylocentrotus purpuratus	Purple sea urchin	NM_001025209.1
Lingula anatina	Lingulata	XM_024076007.1
Crassostrea gigas	Pacific oyster	XM_011451881.2
Mnemiopsis leidyi	Sea walnut	ML29314a

Heliconius melpomene	Postman butterfly	HMEL015715
Danaus plexippus	Monarch butterfly	DPOGS208170
Bombyx mori	Silkworm	XM_021351392.1
Anopheles gambiae	Mosquito	AGAP008683
Ectocarpus silliculosus	Brown algae1	CBJ26102.1
Ectocarpus silliculosus	Brown algae2	CBJ29407.1
Auratiochytrium limacinum	Labyrinthulomycete1	Aurli1|36553
Auratiochytrium limacinum	Labyrinthulomycete2	Aurli1|3394
Auratiochytrium limacinum	Labyrinthulomycete3	Aurli1|29869
Auratiochytrium limacinum	Labyrinthulomycete4	Aurli1|11945
Allomyces macrogynus	Fungi1	**AMAG_15879**
Allomyces macrogynus	Fungi2	**AMAG_14269**
Salpingoeca rosetta	Apunsozoa	XM_004990475.1
Thalassiosira pseudonana	Diatom	XM_002289477.1
Dictyostelium discoideum	sGC_Amobea	XM_638127.1
Thecamona trahens	Choanoflagellate1	XM_013900106.1
Thecamona trahens	Choanoflagellate2	XM_013898968.1
